# Relict or reintroduction? Genetic population assignment of three Tasmanian devils (*Sarcophilus harrisii*) recovered on mainland Australia

**DOI:** 10.1098/rsos.170053

**Published:** 2017-04-26

**Authors:** Lauren C. White, Jeremy J. Austin

**Affiliations:** Australian Centre for Ancient DNA, School of Biological Sciences, University of Adelaide, North Terrace, Adelaide, South Australia 5005, Australia

**Keywords:** Tasmanian devil, relict population, Lazarus species, extinction

## Abstract

Today, the Tasmanian devil (*Sarcophilus harrisii*) is found only on the island of Tasmania, despite once being widespread across mainland Australia. While the devil is thought to have become extinct on the mainland approximately 3000 years ago, three specimens were collected in Victoria (south-eastern Australia) between 1912 and 1991, raising the possibility that a relict mainland population survived in the area. Alternatively, these devils may have escaped captivity or were deliberately released after being transported from Tasmania, a practice that has been strictly controlled since the onset of devil facial tumour disease in the early 1990s. Such quarantine regimes are important to protect disease-free, ‘insurance populations’ in zoos on the mainland. To test whether the three Victorian devils were members of a relict mainland population or had been recently transported from Tasmania we identified seven single nucleotide polymorphisms (SNPs) in the mitochondrial genome that can distinguish between Tasmanian and ancient mainland populations. The three Victorian devil specimens have the same seven SNPs diagnostic of modern Tasmanian devils, confirming that they were most likely transported from Tasmania and do not represent a remnant population of mainland devils.

## 1. Introduction

Lazarus taxa—species thought to be extinct but then rediscovered alive—stand in stark contrast to the current worldwide extinction crisis. The Australian vertebrate fauna has suffered a high rate of anthropogenic extinctions in the last 200 years, but also has a relatively high rate of species rediscovery [1]. Australian Lazarus species include the night parrot (*Pezoporus occidentalis,* rediscovered in 1990 after no sightings for almost 80 years) [2], the Dibbler (*Parantechinus apicalis*, presumed extinct in the wild for 63 years until an individual was captured in 1967) [3], Gilbert's potoroo (*Potorous gilbertii* rediscovered alive in 1994 after having been thought extinct for over 100 years) [4] and the tiger quoll (*Dasyurus maculatus,* observed on a motion-detecting camera in the Victorian Grampians in 2013, 141 years after it was last seen in that area) [5]. The rediscovery of a number of small to medium-sized mammals and birds in recent decades raises the possibility that other species have survived undetected.

The Tasmanian devil is a possible example of a Lazarus species previously thought to be extinct for thousands of years in part of its former range. Devils are the largest extant carnivorous marsupials and are currently found only on the island of Tasmania. Along with the thylacine (*Thylacinus cynocephalus*), devils were once widespread across the Australian mainland, but both species are thought to have become extinct there during the mid-Holocene (approx. 3000 years before present; [6,7]). More recent dates (430–630 years before present) for devil remains on the mainland have been rejected on methodological grounds [6].

Although early Europeans did not record living devils on the mainland, three devil specimens were collected in Victoria, in the southeast of the Australian mainland, between 1912 and 1991, two of which were found alive [8–10]. These discoveries raise the intriguing possibility that devils were not extinct on the mainland at the time of European arrival and that a remnant population persisted in Victoria until modern times. Recent examples of Lazarus species in Australia lend credibility to the idea that a relict devil population could have survived in the south-eastern corner of the mainland, a bioregion that resembles that of Tasmania [11]. Alternatively, the recovered Victorian devils may represent animals transported from Tasmania that were kept as pets or in wildlife parks, that subsequently escaped or were deliberately released. Museum specimens of all three animals are held at Museum Victoria, offering the opportunity to use preserved DNA to identify their origin.

Identifying the origins of the Victorian devil specimens has broader relevance than a simple natural history investigation. Understanding trends in wildlife trade and translocation are important for the design of quarantine regimes, which have become very important for Tasmanian devils since the onset of devil facial tumour disease (DFTD), a highly contagious cancer that has wiped out over 80% of the population [12]. The transport of devils out of Tasmania is now strictly controlled to ensure that ‘insurance populations’ of disease-free devils on the mainland are not threatened by DFTD. There are also scientific and public calls for devils to be translocated to the mainland as part of a rewilding programme to restore ecological functions to ecosystems dominated by introduced predators [11]. To date, none of these proposals have considered the impacts of translocating Tasmanian devils into areas potentially already occupied by relict populations of mainland devils. Therefore, for both insurance population management and rewilding proposals, it is critical to know if a remnant population of devils survives on the mainland, and whether this population represents an ancient relict or a recent introduction. To test whether the Victorian devils were members of a relict mainland population or had been recently transported from Tasmania, we analysed and compared mitochondrial DNA from the three museum specimens with data from Tasmanian (extant and subfossil) and mainland (subfossil) devils.

## 2. Material and methods

### 2.1. Samples and DNA extraction

**Figure 1.**
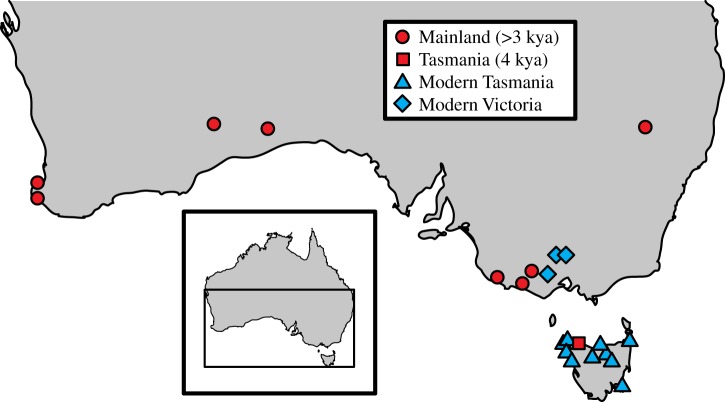
Map of southern Australia showing the collection locality for ancient mainland, modern Tasmanian and ancient Tasmanian samples used to identify geographically informative single nucleotide polymorphisms (SNPs), and the location of the three modern Victorian devil specimens of unknown origin.

Three devil specimens were collected in central Victoria between 1912 and 1991 and subsequently donated to Museum Victoria (figure 1, table 1). C6257 was caught and killed by a farmer in 1912 [9], C31255 was collected in 1991 and has skeletal damage that is consistent with road kill, and C22543 was caught alive in a rabbit trap in 1971 [10]. Samples of toepad (C22543), dried tissue (C31255) or tooth (C6257) were taken from the specimens and transferred to the Australian Centre for Ancient DNA (ACAD), at the University of Adelaide. We controlled for contamination of the museum samples with contemporary DNA and previously amplified mitochondrial DNA PCR products by conducting all pre-PCR work in a dedicated, physically separate, cleanroom facility, with the use of dead-air glove boxes fitted with internal ultraviolet lights; regular decontamination of all work areas and equipment with sodium hypochlorite; personal protective equipment, and strict one-way movement of personnel. No contemporary devil samples or DNA had ever been present in the pre-PCR laboratory and we included a negative control alongside all extractions to monitor for contamination. DNA was extracted using the DNeasy Tissue Kit (Qiagen) according to the methods described by Boessenkool *et al.* [13] (tooth) and Austin *et al.* [14] (tissue and toepad).

**Table 1.** Museum specimen details for three Tasmanian devils collected in Victoria.

Museum Victoria accession number	date collected	specimen type	sex	locality	lat.	long.
C6257	1912	skin and skeleton	female	Tooborac	−37.05	144.78
C31255	28 March 1991	skin and skeleton	female	Faraday	−37.05	144.30
C22543	22 May 1971	skin	male	Dereel	−37.82	143.75

### 2.2. Primer design and amplification

**Figure 2.**
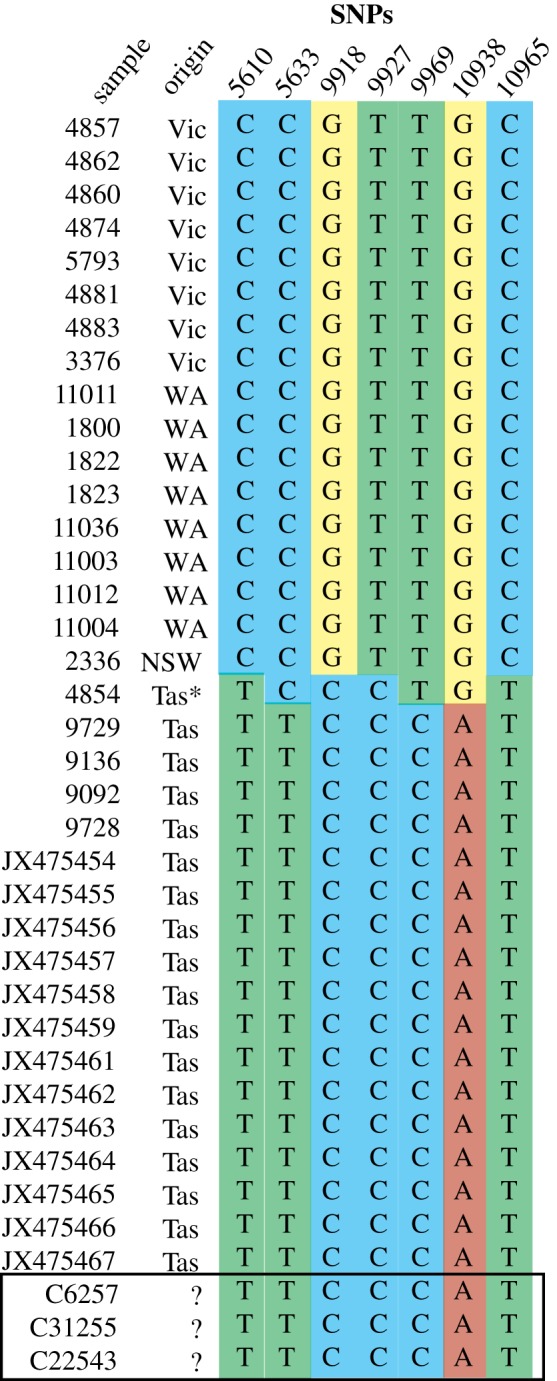
DNA sequence variation at seven mtDNA SNPs in 17 mainland and 18 Tasmanian devils, and the three Victorian devils of unknown origin (highlighted by box). Asterisk indicates an ancient Tasmanian sample approximately 4000 years old. SNP numbers refer to nucleotide position in the devil mitochondrial genome, GenBank Accession: JX475463. Sample numbers 2336–11036 are ACAD sample numbers, JX475454–JX475467 are GenBank Accession numbers from [15].

An alignment of 35 devil mitochondrial genomes including 18 Tasmanian, and 17 sub-fossil mainland samples (13 Tasmanian samples from [15], five Tasmanian and 17 mainland samples from A. Brüniche-Olsen *et al*., unpublished data) was used to identify seven informative single nucleotide polymorphisms (SNPs) (figure 2) that distinguish between mainland devils, contemporary Tasmanian devils, and an extinct haplotype identified from a single ancient (approx. 4000-year-old) Tasmanian devil collected from Smithton. The mtDNA genomes from Brüniche-Olsen *et al*. (unpublished data) were generated from genomic DNA libraries enriched for mtDNA and sequenced on an Illumina Miseq. SNPs in these mtDNA genomes were called with a mapping quality Phred score >30 and a minimum read depth of 10. The seven diagnostic SNPs are located in the tRNA-Cys (*n *= 2), COX3 (*n *= 3) and ND4 (*n *= 2) genes. Three 63–74 bp fragments (excluding primers) covering the seven SNPs were selected for amplification. Primers were designed from the same alignment using Primer3 in Geneious R7 (Biomatters). Forward and reverse primers for each fragment are (Fragment 1-tRNA-Cys: 5′-GTTCTCTACATAAGCCCTGG-3′, 5′-GGTCTTATTTGAACCTAAGCC-3′; Fragment 2-COX3: 5′-TTGGTTCTCTCTTCCTAAT CG-3′, 5′-TACGAAGTGTCAGTATCAGG-3′; Fragment 3-ND4: 5′-TAGCATTTGAAGCTTAACCC-3′, 5′-AGTCAGCAGGATAGGATAAG-3′).

Singleplex PCRs were performed in 25 µl volumes containing 0.5 U Platinum Taq Hifi, 1x Platinum Hifi buffer, 0.25 mM each dNTP, 2 mM MgSO_4_, 0.25 mg ml^−1^ Rabbit Serum Albumin, 0.4 µM forward primer, 0.4 µM reverse primer and 2 µl of sample DNA. Thermocycling consisted of 94°C for 2 min followed by 40 cycles of 94°C for 15 s, 55°C for 15 s, 68°C for 30 s, and a final extension of 68°C for 10 min. All PCRs included a negative no-template control to check for cross-sample contamination and the extraction negative control. Amplification success was determined by running 2.5 µl of product on 3.5% agarose gels stained with GelRed (Biotium, Hayward, USA). Positive amplification products were Sanger sequenced in both directions using BigDye chemistry and an Applied Biosystems 3730xl DNA Analyzer at the Australian Genome Research Facility (Adelaide, Australia).

### 2.3. Sequence alignment and population assignment

Sequence chromatograms were trimmed for low quality data (error probability limit = 0.05) and the reads assembled to a reference devil mitogenome (JX475463) in Geneious 7 (Biomatters) using the high sensitivity option. Ambiguities were visually inspected and consensus sequences for each of the three individuals were aligned to the original 35 Tasmanian and mainland sequences.

## 3. Results and discussion

We obtained 203 bp of mtDNA sequence, including the seven population-informative SNPs, for the three devil specimens recovered from Victoria. All three samples matched the contemporary Tasmanian devils at all seven SNPs (figure 2). These results suggest that the devils recovered in Victoria originated from Tasmania and do not represent a relict mainland population. The consensus of fossil evidence, suggesting that mainland devils became extinct approximately 3000 years ago [6,7], support these results. These animals may have been kept as pets or in private zoos, and they may have subsequently escaped or been released. A newspaper report [10] describing the quiet nature of the Dereel animal (C22543) captured in 1971 is consistent with a significant part of its life spent in captivity. At least two devils have been recorded as escaping from Victorian wildlife parks—one in 1939 from the Ballarat Zoological Gardens [16] and the second in 2009 from the Maru Koala Park near Grantville. There are no reports that the Ballarat animal was re-captured but a roadkill devil was found close to the Maru Koala Park a short time after it went missing [17].

Animal translocation and trade is a major contributor to the spread of disease [18] and is consequently a significant concern for the Tasmanian devil since the onset of DFTD. To guard against total species extinction, a number of disease-free, captive insurance populations have been established on mainland Australia [19]. Additionally, there have been an increasing number of calls to reintroduce devils into the mainland wild, not only as a precaution against extinction, but also as a method to suppress introduced foxes and cats that are devastating many native animal populations [11,20]. Our results emphasize that animals kept in captivity may escape or be released, which highlights the importance of identifying and controlling wildlife trade and translocation. Given the precarious nature of the Tasmanian devil in the wild, the strict quarantine regimes are important for mainland insurance populations and may become even more so if reintroduction to the mainland is considered.

## Data accessibility

DNA sequences for the seven diagnostic SNPs for all devil samples included in this study are available in figure 2.
